# Breastfeeding Education Across the Perinatal Period: A Descriptive Exploratory Pre-Implementation Study

**DOI:** 10.34763/jmotherandchild.20263001.d-26-00019

**Published:** 2026-09-25

**Authors:** Jennifer Abbass-Dick, Adrianna Trifunovski, Aisha Hussain, Manon Lemonde, Catherine Shaw, Keri Durocher

**Affiliations:** University of Ontario Institute of Technology, Faculty of Health Sciences, Oshawa, Ontario, Canada; ELM Avenue Management, Toronto, Ontario, Canada; Sheridan College, School of Nursing, Brampton, Ontario, Canada

**Keywords:** Mothers, Breastfeeding, Education, Implementation, Digital Health, Experiences

## Abstract

**Background:**

Breastfeeding is the optimal infant feeding method; however, cessation rates remain high across many developed nations. Support for mothers can be improved through breastfeeding education that spans the entire perinatal period. Due to the number of healthcare providers involved, delivery of breastfeeding education must be consistent and tailored to individual needs. Digital innovations are a potential solution to enhance accessibility and consistency in breastfeeding education delivery. How to best implement these solutions in clinical settings is not yet known.

**Materials and methods:**

We used a non-experimental, exploratory descriptive survey to collect mothers’ and providers’ experiences of breastfeeding education, and their perceptions of how an eHealth resource can be adapted and implemented to standardise breastfeeding education.

**Results:**

The surveys were completed by 93 providers and 78 mothers. Mothers reported receiving education from diverse providers across the perinatal period, with varying information at different time points. Information was received on many topics that changed at various times. Oral communication was ranked as the most frequent education method. Both groups reported that online videos and websites would be the most effective digital solutions to support breastfeeding education.

**Conclusion:**

Providers play a crucial role in supporting mothers through education delivery that spans the entire perinatal period. By integrating the findings, accessibility and consistency of breastfeeding education could be enhanced by implementing the eHealth resource across organisations in a health region.

## Introduction

Due to the abundant benefits breastfeeding provides for mothers and infants, global healthcare organizations promote it as the most optimal feeding method for the first two years and beyond [1]. The Baby-Friendly Hospital Initiative (BFHI) is a global program that was created through a collaboration between the World Health Organization (WHO) and the United Nations Children’s Fund to promote and support breastfeeding within healthcare organisations (hospitals, public health facilities, etc.). This initiative includes the “Ten Steps to Successful Breastfeeding”, a written policy with clinical guidelines for healthcare organisations to follow to uphold the BFHI. The BFHI has a wide global reach — A recent WHO assessment demonstrated 86% of surveyed countries had various organisations implementing the BFHI [2]. Despite the global reach of the BFHI, breastfeeding rates remain suboptimal in many developed countries [3]. For example, in Canada, only about one out of every three women exclusively breastfeed their infants for the first six months of life. This percentage in the United States is even lower, with a rate of approximately one in every four women breastfeeding exclusively for six months [4].

### Breastfeeding education and support

One notable barrier to exclusive and long-term breastfeeding is a lack of education and support for mothers and their families, both within the immediate postpartum period and within the community after hospital discharge (even after delivering in BFHI-certified settings) [5,6]. Results from multiple studies have demonstrated that mothers reported negative and stressful experiences while trying to establish breastfeeding, and they are surprised when it does not happen easily [7,8,9]. There is also a lack of knowledge amongst mothers about where to access credible breastfeeding information [7,9], and the information that is accessed has been reported as insufficient or inconsistent [7,8,9,10].

### Digital health solutions

Mothers encounter a variety of healthcare providers (HCPs) in different organisations through the perinatal period, from conception to postpartum recovery [8,11,12]. Systematic review findings have demonstrated that consistent breastfeeding education should be repeated at least four times in the perinatal period to mothers and their family members and should continue during hospital admission and in the community after discharge [13,14,15]. Digital innovations for breastfeeding support are increasingly implemented as a solution to enhance accessibility for both HCPs and mothers [13,16,17]. For example, Almohanna et al. (2020) conducted a systematic review where they found that a variety of digital tools are utilised for breastfeeding support, such as mobile phone apps, online discussion forums, and web-based consultation.

Some interventions are now targeted at specific populations. For example, Lewkowitz et al. (2021) conducted a randomised, controlled trial (RCT) to examine the results of a smartphone application for low-income, first-time mothers and found that the intervention group had a higher rate of reporting their breastfeeding challenges via the app [17]. Additionally, Huang et al. (2024) conducted an RCT to determine whether a digital co-parenting intervention led to increased breastfeeding outcomes [18]. Mothers who had access to the co-parenting intervention had significantly higher breastfeeding duration, knowledge, and feelings of competence. As more digital resources become available, the standardisation of materials that deliver consistent information about breastfeeding should be of the utmost importance[10].

### Purpose

Our study team has created and evaluated eHealth resources tailored to the breastfeeding needs of specific populations, including couples [19], Indigenous families [20], and young mothers [21]. We found that access to these resources resulted in high breastfeeding rates in both study groups in the RCTs conducted with mothers and their co-parents in Canada [22]. Additionally, Mothers have indicated the number one source of breastfeeding information is websites [22].

The results of these studies led to an implementation project designed to standardise breastfeeding education using an eHealth resource in clinical interactions in healthcare organisations. The project was carried out across a health region in Ontario, Canada, specifically a local health unit and three hospitals that provide perinatal care. HCPs also completed surveys, which showed that adaptations were required to implement the eHealth resources in practice [23]. The HCPs specifically wanted to determine how this digital resource could be used in clinical settings during face-to-face interactions.

To develop the implementation plan, the intervention needed to be adapted to the local context and embedded within policies and procedures. Therefore, the who, what, when, where and how of breastfeeding education in clinical settings had to be determined [24,25]. The purpose of the pre-implementation survey was to collect feedback from mothers and HCPs about how breastfeeding education is delivered and to determine how to best integrate the eHealth resource into organisational policies and procedures. We also wanted to determine how to best adapt the technology, the eHealth resource, for use in face-to-face clinical interactions. HCPs and parents were both included in this study as they are the key stakeholders and end users in this implementation project, and their input into the design of the implementation plan is critical to its success. Additionally, to determine who, what, when, where, and how breastfeeding education is delivered, both perspectives were necessary.

## Materials and methods

In this pre-implementation study, we used a non-experimental, exploratory descriptive survey for data collection. To recruit potential participants, posters were shared via a Listserv and email to Ontario-based organisations for HCPs that work with families during the perinatal and breastfeeding periods. The health care organisations shared the recruitment poster on their social media channels and in their offices with parents. Parents with a child three years of age and younger in Ontario, Canada were invited to complete an online survey regarding the breastfeeding education they received. The surveys were designed to identify when, where, how, and who provided education to inform the eHealth resource implementation plan and to adapt the resource to the local context. HCPs and parents had separate online surveys that covered the same topics and collected data from different perspectives. The survey data collection took place from April to August 2024. Eligible mothers had a child within the past three years, and eligible HCPs worked with perinatal families. After completing the online consent form, participants used Google Forms to complete the surveys, which included both closed and open-ended questions. Closed-ended questions elicited information from HCPs on 1) their role in delivering breastfeeding education (in what professional capacity, in what setting, at what point in the perinatal period, etc.) 2) at what point should breastfeeding education be delivered, 3) topics covered and considered most important, 4) methods of information delivery used, 5) what they would like to see created to deliver breastfeeding education. Parent surveys included questions related to 1) demographic information, 2) when and who delivered breastfeeding education across the perinatal period, 3) what topics were covered at different time points and perceived to be most important, and 4) what information delivery method was used to provide information and is preferred. Open-ended questions allowed participants to provide additional feedback and suggestions related to breastfeeding education and what resources could be created to aid in delivering this information in clinical settings. The analysis of qualitative findings from open-ended questions is published elsewhere. This work was reviewed by the University’s Research Ethics Board [REB #17677].

## Results

### Participant characteristics

The surveys were completed by 93 HCPs. Most HCPs worked in community/outpatient settings (n=66, 71.0%), fewer worked primarily in hospitals (n=10, 10.8%), and some worked in both settings (n=17, 18.3%). The HCPs held a wide range of professional roles, most commonly public health nurses (n=53, 57%) and lactation consultants (n=31, 34%), followed by nurses, midwives, registered dietitians, prenatal educators, and physicians. HCPs had substantial experience working with perinatal families, with most having more than ten years of experience. (Table 1).

**Table 1.** Health Care Providers’ Demographics.

**Category**	**Response Item**	**N=93 N (%)**
Work Setting	Community	66 (71.0)
	Hospitals	10 (10.8)
	Both community and hospitals	17 (18.3)
Years of Work Experience	1-10	1-10 – 37 (39.8)
	11-20	11-20 – 36 (38.7)
	21-30	21-30 – 14 (15.1)
	> 30	> 30 -4 (4.3)
	No response	-2 (2.2)
Professional Role	Public health nurse	53 (57)
	Lactation consultant	31 (34)
	Nurse	18 (19.4)
	Midwife	4 (4.3)
	Registered dietician	5 (5.7)
	Prenatal educator	4 (4.5)
	Peer lactation counsellor	4 (4.5)
	Family physician	3 (3.2)
	Doula	2 (2.3)
	Nurse practitioner	1 (1.1)
	Chiropractor	1 (1.1)
	Naturopathic doctor	1 (1.1)
	Other	3 (3.2)

The survey was completed by 78 parents who all identified as mothers. Their youngest child ranged in age, with the largest age category being < 24 months, and a majority had one or two children (91%) (Table 2). These mothers were asked to provide information on their breastfeeding experience in an open-ended question. All mothers indicated they had experience with breastfeeding, with 41% reporting breastfeeding for longer than 12 months, 16.7% reporting combination feeding or supplementation, and 11.5% reporting feeding human milk via bottle (6.4% doing so exclusively).

**Table 2.** Mothers’ Demographics.

**Demographic**		**N=78 N (%)**
Parenting role	Mother	78 (100)
	Father	0
	Partner	0
	Birth Parent	0
Age of youngest child	0-< 6 months	13 (16.7)
	6 months-< 12 months	13 (16.7)
	12 months< 18 months	15 (19.2)
	18 month< 24 months	13 (16.7)
	> 24 months	24 (30.8)
Number of children	1	39 (50.0)
	2	32 (41.0)
	3	6 (7.7)
	4	1 (1.4)

### Maternal response to when and who provided education

**Figure 1.**
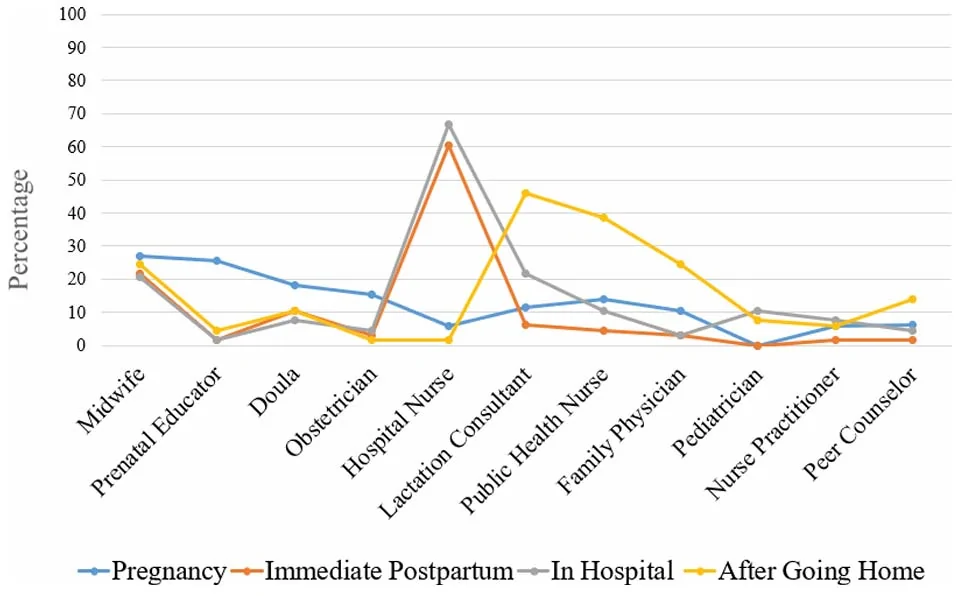
HCPs That Provided Breastfeeding Education Over Perinatal Period.

Mothers were asked who they had received breastfeeding education from at varying time points. They reported receiving education from a wide range of HCPs across the perinatal period (Figure 1). During pregnancy, breastfeeding information was provided mostly by midwives (27%) and prenatal educators (26%), followed by doulas, obstetricians, public health nurses, and lactation consultants. Whereas, in the postpartum period, breastfeeding information was mostly provided by hospital nurses (68%), followed by lactation consultants (23%) and midwives (22%). Post hospital discharge, the pattern shifted, with lactation consultants reported most frequently as information providers (47%), followed by public health nurses (38%), family physicians (24%), and midwives (24%). Some mothers reported receiving breastfeeding information from paediatricians, nurse practitioners, or peer counsellors during this period. Across the perinatal period, midwives had the most consistent involvement in breastfeeding education. However, midwifery care is discontinued around six to eight weeks postpartum [26].

### HCP responses to when and who should provide breastfeeding education

**Figure 2.**
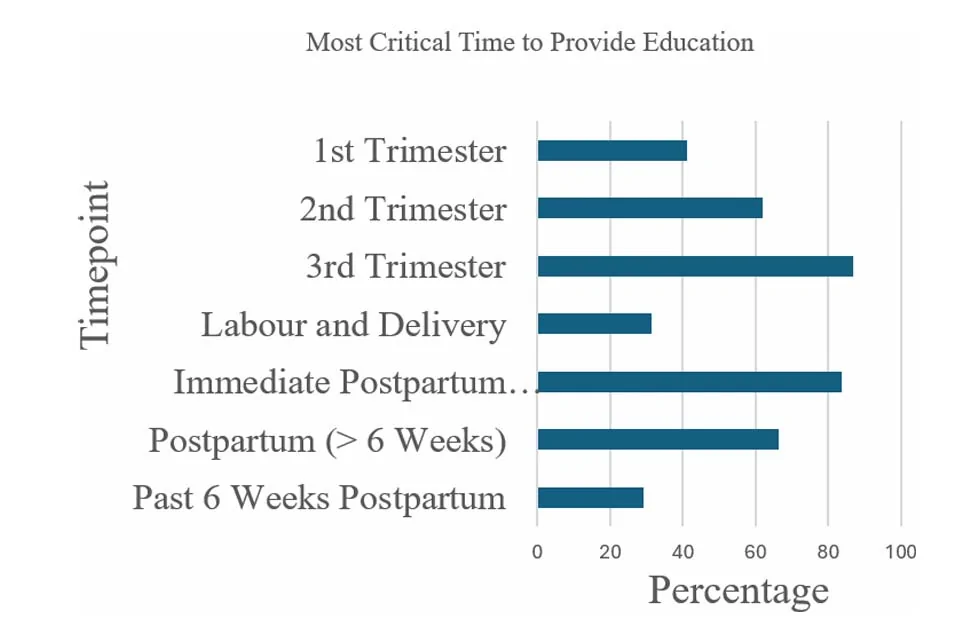
Most Critical Time to Provide Breastfeeding Education.

HCPs were asked when breastfeeding education should be provided. Their responses indicated how education should be delivered throughout the entire perinatal period, from prenatal to past six weeks postpartum (Figure 2). The most frequently reported time periods were the third trimester of pregnancy (87.0%), followed by the immediate postpartum period (24–48 hours) (83.7%).

HCPS were asked which providers should be responsible for delivering breastfeeding education, and their responses indicated a diverse group across the perinatal period. These multidisciplinary HCPs should work together to provide consistent information to mothers. One HCP stated, “*Midwives, obstetricians/gynocologists, lactation consultants, family doctors, public health nurses; We all need to work together to support moms*” [HCP Participant #70]. Although many HCPs work with mothers during this time, there is a difference in the knowledge and skills to support breastfeeding. Therefore, a system for referrals to more specialised lactation professionals is needed. As one HCP suggested: “*Family physicians/obstetricians/midwives seeing families prenatally first with at least where to find more information (directing them to websites or breastfeeding classes they can attend), then the nurses or midwives at the birth and postpartum, followed by lactation consultants when appropriate*” [HCP Participant #82]. Another HCP stipulated, “*Only providers with current and ongoing breastfeeding education. Otherwise, they should refer out*” [HCP Participant #25].

### Important breastfeeding topics to be covered

**Figure 3.**
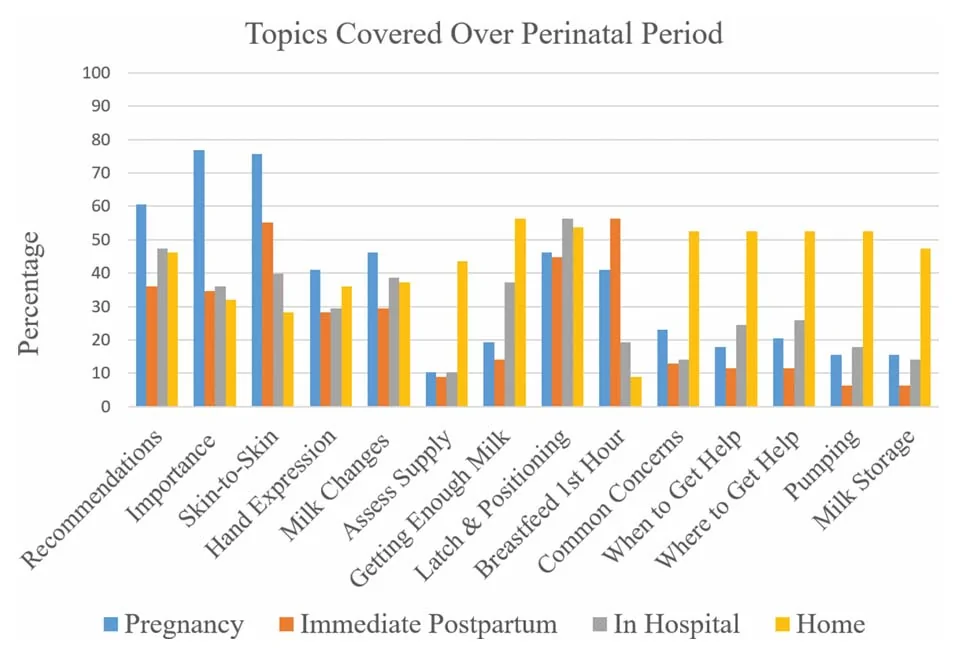
Topics Covered Over Perinatal Period.

Mothers reported receiving information on a wide range of breastfeeding topics across the perinatal period (Figure 3). The most frequently discussed topics changed at different periods of time. The topics discussed in pregnancy and immediately postpartum most often aligned with the ones mothers indicated as most important to learn about during those time points. In pregnancy, these included the importance of breastfeeding to the mother and infant, importance of skin-to-skin contact, and breastfeeding recommendations (Table 3). Immediately postpartum, the topics were breastfeeding within the first hour after birth, importance of skin-to-skin contact, and mechanics of breastfeeding. In hospital, the importance of information on the mechanics of breastfeeding remained consistent; however, other frequently covered topics were breastfeeding recommendations and the importance of skin-to-skin contact. Additional topics mothers indicated as most important in pregnancy and immediately postpartum included how to know if the baby is getting enough milk and where to get help. After discharge, breastfeeding education shifted toward support, and the topics covered most often by HCPs aligned with those the mothers found the most important. These topics included how to know if the baby is getting enough milk, common concerns, the mechanics of breastfeeding, and when and where to get help.

**Figure 4.**
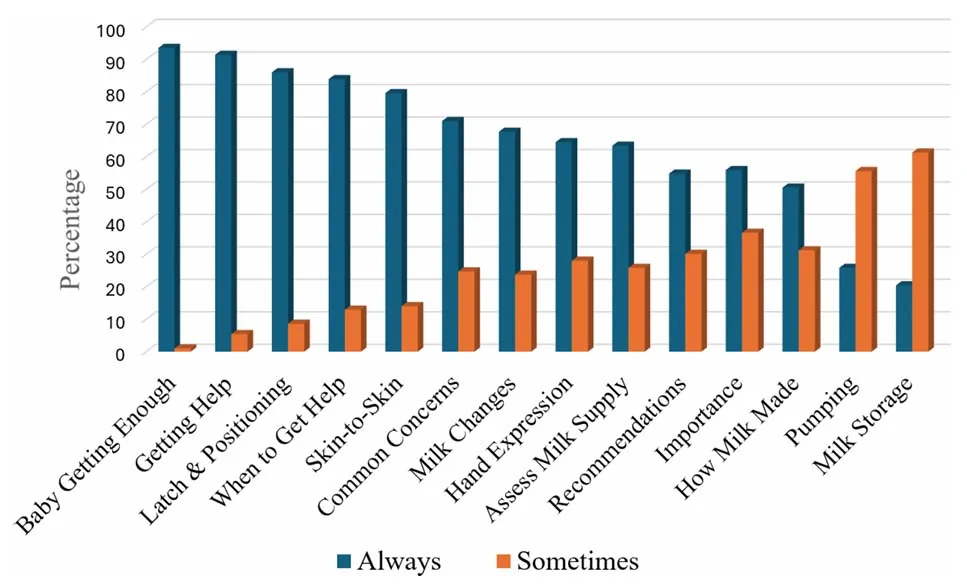
Topics Discussed by HCPs.

HCPs were also asked which topics they consistently provide education on across the perinatal period (Figure 4). The most commonly covered topics included how to know if the baby is getting enough milk (n=87, 93.5%), where to get help (n=85, 91.4%), mechanics of breastfeeding (n=80, 86.0%), when to get help (n=78, 83.9%), and common concerns (n=66, 71.0%) (Table 3). When asked which topics to include in the eHealth resource, HCPs recommended latch and positioning, milk production and maintenance, where to get help, how to know when the baby is getting enough milk, what to expect, and supplementing/pumping/hand expression/formula feeding.

**Table 3.** Breastfeeding Education Topics.

**Topic**	**Parent (n=78)**								**HCP (n=93)**	

	**Pregnancy**		**Immediately post birth**		**In hospital**		**After going home**			
				
	**Provided n (%)**	**Most important n (%)**	**Provided n (%)**	**Most important n (%)**	**Provided n (%)**	**Most important n (%)**	**Provided n (%)**	**Most important n (%)**	**Always provided n(%)**	**Most important n (%)**
Breastfeeding recommendations	47 (60.3)	45 (57.7)	28 (35.9)	15 (19.2)	37 (47.4)	24 (30.8)	36 (46.1)	23 (29.5)	51 (54.8)	28 (30.1)
Importance of BF to mom and babe	60 (76.9)	53 (67.9)	27 (34.6)	19 (24.2)	28 (35.9)	19 (24.2)	25 (32)	13 (19.6)	52 (55.9)	9 (9.7)
Importance of skin to skin	59 (75.6)	50 (64.1)	43 (55.1)	43 (53)	31 (39.7)	24 (30.8)	22 (28.2)	14 (17.9)	74 (80)	14 (15)
Hand expression	32 (41.0)	32 (41.0)	22 (28.2)	23 (23.5)	23 (29.5)	22 (28.2)	28 (35.9)	14 (17.9)	60 (64.5)	15 (16.1)
Milk changes in the first few days	36 (46.2)	26 (33.3)	23 (29.5)	22 (28.6)	30 (38.5)	30 (38.5)	29 (37.2)	14 (17.9)	63 (67.7)	-
How to assess your milk supply	8 (10.3)	23 (29.5)	7 (9)	18 (23.1)	8 (10.3)	28 (35.9)	34 (43.6)	30 (38.5)	59 (63.4)	25 (26.9)
How to know your baby is getting enough	15 (19.2)	30 (38.5)	11 (14.1)	29 (37.2)	29 (37.2)	49 (62.8)	44 (56.4)	40 (51.3)	87 (93.5)	14 (15)
Mechanics-latch position suck swallow	21 (26.9)	35 (44.9)	32 (44.8)	46 (59.0)	44 (56.4)	52 (66.7)	42 (53.8)	35 (44.9)	80 (86)	66 (71)
Breastfeeding in the first hour after birth	32 (41.0)	35 (44.9)	44 (56.4)	38 (48.7)	15 (19.2)	10 (12.8)	7 (9.0)	6 (7.7)	-	9 (9.7)
Common Concerns	18 (23.0)	25 (32)	10 (12.8)	13 (19.6)	11 (14.1)	31 (39.7)	41 (52.6)	36 (46.2)	66 (71)	5 (7.5)
When to get help	14 (17.9)	32 (40.1)	9 (11.5)	14 (17.9)	19 (24.4)	33 (42.3)	41 (52.6)	31 (39.7)	78 (83.9)	10 (10.8)
Where to get help	16 (20.5)	35 (44.9)	9 (11.5)	16 (20.5)	17 (25.8)	40 (51.3)	41 (52.6)	39 (50)	85 (91.4)	28 (30.1)
Pumping	12 (15.4)	17 (21.8)	5 (6.4)	7 (9)	14 (17.9)	28 (35.9)	41 (52.6)	32 (40.1)	24 (25.8)	-
Milk Storage	12 (15.4)	19 (24.2)	5 (6.4)	8 (10.3)	11 (14.1)	28 (35.9)	37 (47.4)	31 (39.7)	19 (20.4)	-
Other	-	-	-	-	-	-	-	-	-	5 (5.4)

### Breastfeeding information delivery methods

HCPs were asked what information methods they use to provide breastfeeding information. The most common method was oral communication (n=91, 97.8%), followed by online information (n=70, 75.3%), online videos (n=66, 71%), physical props (n=58, 62.4%), booklets (n=57, 61.3%), and pamphlets (n=43, 46.2%).

**Figure 5.**
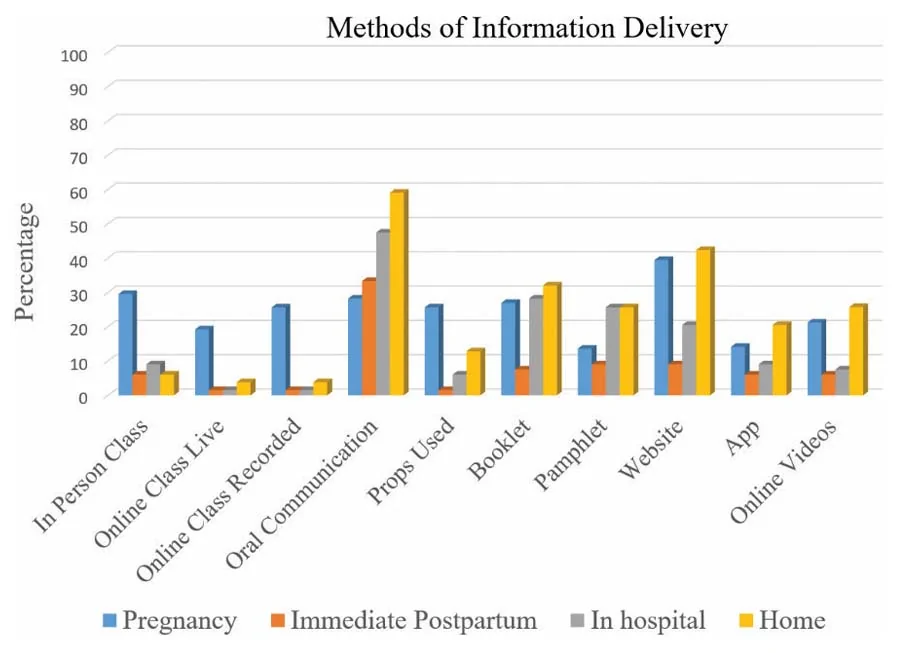
Methods of Information Delivery.

Mothers were asked about the delivery methods used for breastfeeding education at the different time points in the perinatal period. They reported oral communication as the most frequently used information delivery method (prenatally, n=22, 28.2%; immediately after birth, n=26, 33.3%; in the hospital, n=37, 47.4%; post-discharge, n=46, 59%) (Figure 5). Mothers also reported receiving information mainly through in-person or online classes during pregnancy, while online and print resources were used across the perinatal period, including websites, online apps, videos, booklets, and pamphlets.

### Breastfeeding education materials to be created

HCPs and mothers were asked about what formats they would prefer for education materials. Online videos were the preferred method for both groups (83.7% HCP; 55.4% mother), followed by websites (79.3% HCP; 50% mother) and online apps (51.1% HCP; 51.4% mother). Written materials such as booklets (54.3% HCP; 36.5% mother) and pamphlets (41.3% HCP; 28.4% mother) were selected more often by HCPs than mothers. Mothers more frequently selected props, such as dolls or breasts (23.9% HCP; 45.9% mother).

Preferences for education material formatting should be considered in the design of breastfeeding education materials. Feedback from mothers and HCPs indicated that the materials should also be aligned with BFHI standards: comprehensive, evidence-based, up-to-date, and user-friendly. Additionally, all participants requested to have content available in multiple languages and access to QR codes linking to websites, printable materials (PDFs), short videos, and presentations. Other recommendations included developing posters, visuals, handouts, social media content (Instagram/TikTok), and a webinar series. One mother suggested, *“Create standardised province-wide resources/tools with ownership on keeping them [up] to date. Implement a wide promotion campaign to facilitate common messaging among different HCPs”* [Parent Participant #10]. Furthermore, an HCP indicated, *“Honestly, my biggest wish is that there is standardised education about breastfeeding for HCPs themselves; so much inaccurate information is shared. This information should be part of education programs for anyone who works with perinatal families so that everyone is on the same page, providing the same guidance and directing families to the same resources”* [HCP Participant #58]. Another mother stated, *“The generations these days are not going to read long pieces of text. Good images, videos, and short snippets of information via social media platforms or apps that deliver quick daily messages are a good idea”* [Parent Participant #25]. Designing information for partners, grandparents, siblings, friends, families, and employers was suggested by HCPs and mothers. One HCP indicated, *“There needs to be a public campaign to support breastfeeding families aimed at fathers, grandparents, employers… Breastfeeding [is] the normal and unequalled way to feed infants and young children [HCPs must know] their role in supporting breastfeeding families”* [HCP Participant #43].

The information should be presented factually, so that mothers can obtain the information they need to make informed decisions and not feel like they are being told what to do. *“Receiving support that shares the wide range of potential experiences and potential ways to do things… Giving the range of potential things that may or may not work and not providing judgemental or fear-based responses, [as] women may turn away from advice or support in fear of what they may be told”* [Parent Participant #42].

### Findings to support the implementation plan

The survey findings highlighted several elements to be considered for education delivery and adapting the eHealth resource to the local context. To address the “who, when, and where” of breastfeeding education, information should be provided across the perinatal and breastfeeding periods by all HCPs in all settings working with perinatal families. To address the “what and how”, information should be: 1) available in easy to access and understandable formats, 2) engaging, 3) not overwhelming, 4) designed for families, 5) in resources used across settings, 6) evidence-based, 5) standardised, 6) up-to-date, 7) included in print and digitally linked by a QR code, and 8) included in general education with information on where and from whom to access more specialised, individualised support across the perinatal and breastfeeding periods.

**Image 1.**
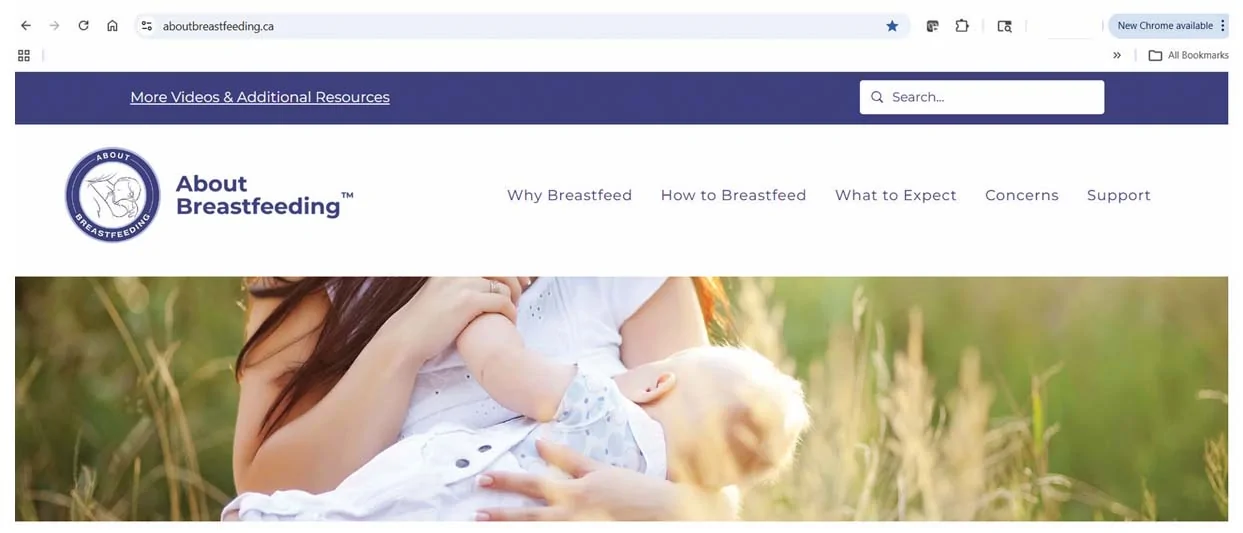
About Breastfeeding Navigation Bar.

**Image 2.**
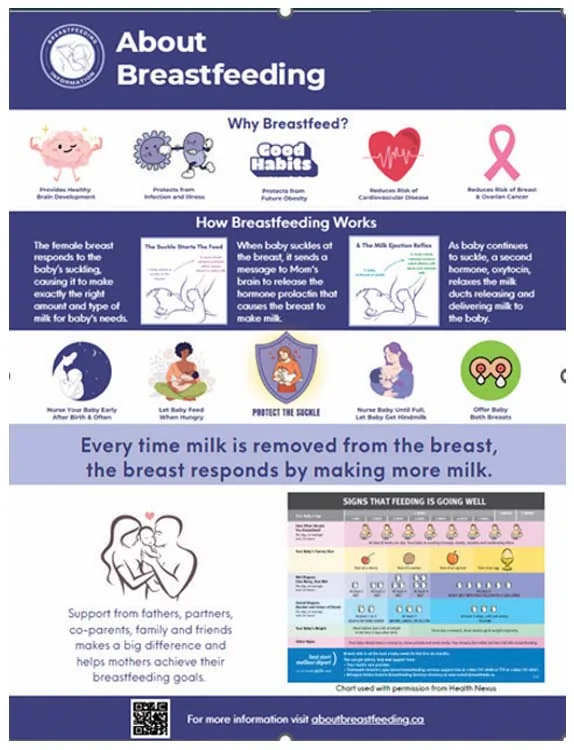
The “About Breastfeeding Summary” Handout.

**Image 3.**
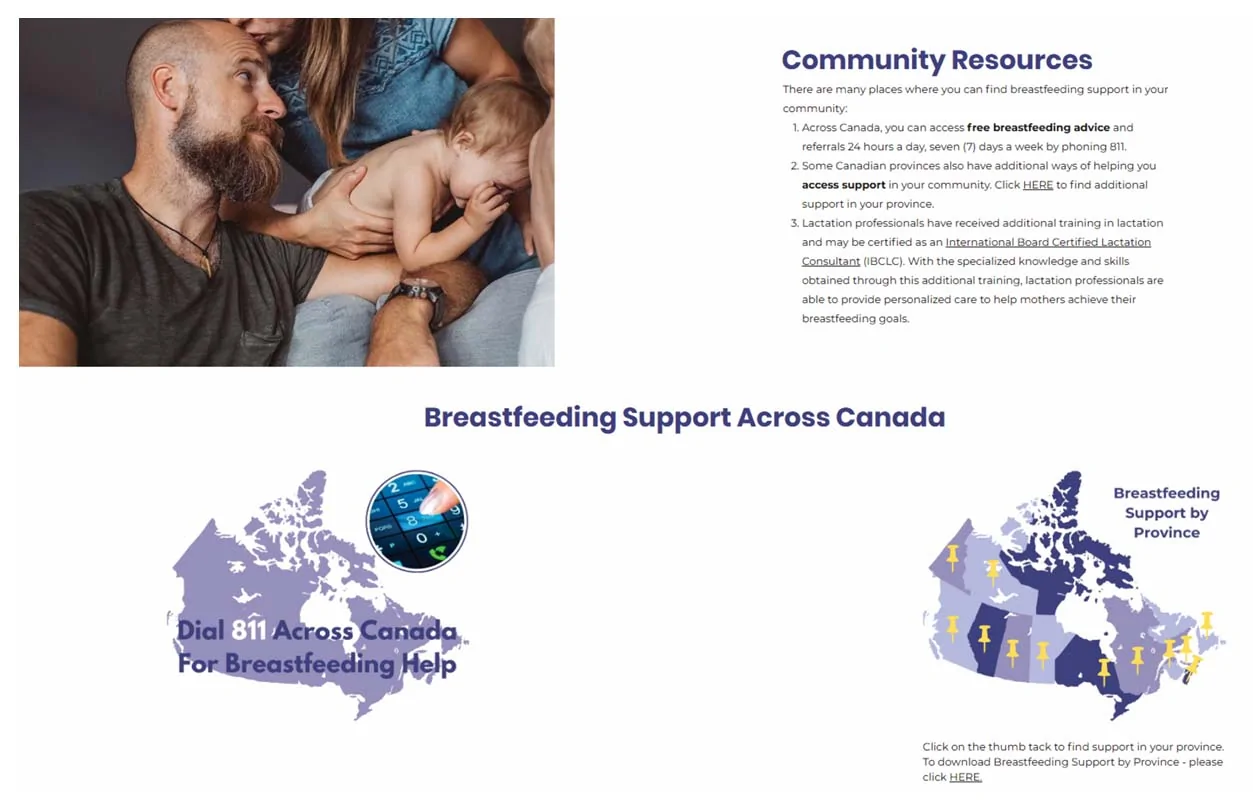
Support webpage information on where to find lactation professionals and assistance across Canada.

To address these recommendations, our team made six main changes to the eHealth resource: 1) A logo and name were designed to be identifiable across formats, linking the print material to the website (About Breastfeeding); 2) The large variety of topics covered in education with parents were collapsed into five key sections to make it easier for users to find information; 3) The website was designed with enhanced navigation and search features, with the sections clearly indicated on the search bar to illustrate the focus of each web page (Image 1); 4) Five handouts with QR codes that linked to the website were created for use in clinical settings (“About Breastfeeding Summary”, “Why breastfeed”, “How to breastfeed”, “Where to find support” handouts, and a threefold Summary pamphlet) (Image 2); 5) Information on the support page was tailored to mothers and family member, providing many tips on how to be involved and work as a team; 6) The support page provides information on where to access personalised, competent lactation support at any time of day and across the perinatal period (Image 3).

## Discussion

This pre-implementation study surveyed HCPs and mothers to determine how breastfeeding information is received across the perinatal period. Breastfeeding education was provided by a variety of HCPs across the perinatal period, with the majority of education delivered orally and in the hospital after delivery. There were a variety of topics covered, and their delivery varied at different time points. Both HCPs and mothers indicated the need for evidence-based and consistent information available in multiple modes so families can make informed decisions about breastfeeding. Lastly, mothers require information on where to access personalised, competent breastfeeding support to address families’ specific needs. This information will be used to make adaptations within local contexts to ensure the eHealth resource can assist HCPs in providing consistent, engaging, and accessible breastfeeding information to families across the perinatal period and for the duration of their breastfeeding journeys.

The results of this study demonstrate the diverse group of HCPs, as the participants represented eleven different professional roles that deliver breastfeeding education to parents. The range of roles highlights the importance of utilizing a comprehensive, evidence-based resource for consistent information. The HCPs within the study echoed this concern and the importance of consistency in providing comprehensive education tailored to the needs of families. Addressing the inconsistencies about breastfeeding information is critical, as mothers can lose trust in HCPs and not seek additional support [27,28]. Education is needed as mothers often think breastfeeding should come naturally and when they experience difficulties, they may lose faith in themselves and their ability to breastfeed [29]. HCPs can address needs by providing clear, skill-based information that fits with mothers’ goals and circumstances across the perinatal period [28]. A recent systematic review and meta-analysis produced similar results related to theory-based education for breastfeeding mothers, with those who received evidence-based education reporting higher self-efficacy and longer breastfeeding durations [30].

Our study findings highlight the need to provide education across the perinatal period and in multiple formats, as most breastfeeding education is delivered in the immediate post-partum period orally by nurses. With early discharge from hospital (24–48 hours after birth) for most mothers, it is difficult for nurses to provide mothers with the necessary education, due to the birth recovery process and learning to parent a newborn [29]. To address this issue, education should begin prenatally and include supplemental material to augment oral education [29].

There was also a wide range of reported topics that were discussed across the perinatal period. The importance of these topics varied depending on the perinatal period stage, and other studies have supported the importance of beginning this education prenatally. For example, Oggero et al. (2024) conducted a systematic review where the findings demonstrated how psychological components of prenatal breastfeeding education were linked to higher breastfeeding rates postpartum, such as the importance of breastfeeding for the baby’s health [31]. Shafaei et al. (2020) conducted an RCT where the intervention group was provided a series of prenatal breastfeeding counselling sessions [32]. Higher self-efficacy levels were self-reported in the intervention group after receiving this education. Therefore, comprehensive information on a variety of topics, delivered in ways that are not overwhelming and align with learning preferences, is needed [27]. These methods will assist mothers to understand lactation, meet individualised goals, and access individualised support when needed.

A strength of this pre-implementation study was involving diverse groups of key stakeholders to ensure all users’ perspectives were included in the design of the implementation plan. By thoroughly exploring the experiences and needs of diverse HCPs and mothers across Ontario, we were able to identify where, when, how, and by whom breastfeeding education is delivered. We were also able to identify clinical settings and procedures to include in the implementation plan of the eHealth resource to standardise education in organisations across a health region. Additionally, we were able to identify similarities and differences in mothers’ and HCPs’ needs and perspectives and determine ways to design the plan to meet each group’s unique needs. However, limitations still exist. Firstly, we used a convenience sample, which may not be reflective of the experiences of all HCPs and mothers. The eligible parents had a child within the last three years, and this time frame may result in recall bias. All parent participants identified as mothers and had breastfed, and therefore some diverse feeding experiences may not have been captured, such as those who opted to not breastfeed or individuals who identify as gender diverse. Demographic data was also not collected as it was not planned for the analysis of the descriptive, exploratory study; however, future phases of this implementation study will include a more in-depth review of breastfeeding education and support needs and sociodemographic factors.

### Key points

- As breastfeeding remains the optimal feeding method for infants, it is imperative to understand how to further support mothers and their families in meeting their breastfeeding goals.
- HCPs play a crucial role in supporting mothers with breastfeeding through education delivery that spans the entire perinatal period.
- The development of a comprehensive, accessible digital resource was supported by understanding which HCPs provide breastfeeding education, relevant topics covered at different time points, and current methods of education delivery.
- By integrating the findings into the development of the eHealth resource implementation plan, breastfeeding support will continue to become more accessible to promote both exclusivity and longevity of breastfeeding, and optimal infant health.
